# Can Expanding Cultural Consumption Improve Urban Air Quality? An Analysis Based on China’s Cultural Consumption Pilot Policy

**DOI:** 10.3390/ijerph20010642

**Published:** 2022-12-30

**Authors:** Bo Li, Jicong Yang, Wei Sun

**Affiliations:** 1 School of Management, Tianjin University of Technology, Tianjin 300384, China; 2 Institute of Geographical Sciences and Natural Resources Research, Chinese Academy of Sciences, Beijing 100101, China; 3 College of Resources and Environment, University of Chinese Academy of Sciences, Beijing 100049, China

**Keywords:** cultural consumption, national cultural consumption pilot, urban air quality, DID

## Abstract

As an important reform exploration to promote economic transformation and upgrading in China, can the national cultural consumption pilot policy improve urban air quality? What are the impact paths? Based on a theoretical analysis of the intrinsic mechanism of expanding cultural consumption affecting urban air quality, this paper constructs the DID model with a quasi-natural experiment, namely the national cultural consumption pilot, to assess the impact of expanding cultural consumption on urban air quality. The results show that: expanding cultural consumption has a significant improvement effect on urban air quality, and the emission reduction effect is also increasing year by year; the heterogeneity analysis shows that expanding cultural consumption has a stronger pollution reduction effect in cities north of the Qinling–Huaihe line, and the effect on air quality is more significant in non-resource cities; the mechanism test indicates that government intervention and public participation have a significant moderating role in the emission reduction effect of cultural consumption. In other words, the higher the level of government intervention and the greater the degree of public participation in the cultural consumption pilot, the stronger the pollution reduction effect of expanding cultural consumption. In addition, cultural consumption has an impact on urban air quality mainly through the industrial structure effect and innovation effect. The findings of this study provide policy insights to further promote the emission reduction effect of cultural consumption and promote urban air quality.

## 1. Introduction

Over the past forty years of reform and opening up, China has relied on rapid industrialization and urbanization to significantly increase its economic strength, but the price behind this proud report card is the increasingly serious problems of environment. Air pollution adds to the huge public health expenditure [1], increases poverty in low-income areas [2] and poses a serious threat to sustainable economic and social development [3]. The 2020 China Ecological Environment Status Bulletin shows that 135 of the 337 cities in the country still exceed the air quality standards. Improving air quality and protecting people’s living standards are the important themes in the Chinese government’s governance. In recent years, the Chinese government has placed construction of ecological civilization in an important position of social development, made unprecedented efforts to protect the ecological environment and promotesustainable economic and social development. Ecological and environmental problems are ultimately a matter of development and lifestyle [4]. Therefore, changing the economic development model that breaks the ecological environment and picking up the old and new dynamics is an important means to solve the ecological and environmental problems. As a pillar industry for fostering new impetus for economic development in the new era of China, the cultural industry plays an important role in promoting upgrading of consumption structure and facilitating economic transformation and upgrading. Considering from the supply side, cultural products are different from general material products, and have the characteristics of high energy consumption and high emissions. Production of cultural products has the characteristics of low energy consumption, low pollution, low emissions and reuse, which meet the requirements of ecological civilization construction and sustainable economic development as required in the new era. Considering from the demand side, cultural consumption is spiritual and green consumption, which can effectively reduce the environmental impact of people’s daily activities. Therefore, is there some link between expanding cultural consumption and urban air quality? If expanding cultural consumption does improve air quality, what are the main pathways of influence? Exploring these issues are not only beneficial to better promote the development of cultural industries but also have important practical implications for solving the air pollution challenge.

Air pollution, as an undesirable product of economic development, is inseparable from economic activity. For example, Grossman and Krueger proposed the theory of the environmental Kuznets curve through a study of pollution levels and per capita income [5]. In addition, scholars have proposed the “pollution paradise hypothesis” [6] and the “pollution halo hypothesis” [7,8] in their studies on foreign direct investment and environmental pollution. As research continues, more literature is beginning to analyse the different perspectives of economic development. In their analysis of the spatial heterogeneity of economic development and industrial pollution in Chinese cities, He et al. found an inverted “U”-shaped relationship between economic density and industrial SO_2_ emissions [9]. Danish and Wang found an N-shaped relationship between support income and pollution when they studied the relationship between biomass energy consumption and environmental pollution in the BRICS countries [10]. In addition, there is literature that provides detailed analysis from the perspectives of urbanization [11,12,13], fiscal decentralization [14,15] and industrial agglomeration [16,17,18].

A series of studies have been conducted by scholars on cultural consumption as a new support point for China’s economic transformation and upgrading in the new era. Lu et al. examined the impact of cultural consumption on the quality of China’s economic growth from three perspectives: growth efficiency, stability and sustainability of cultural consumption using inter-provincial panel data in China [19]. Fan studied the impact of cultural consumption on the urban–rural income gap based on the theory of new economic geography [20]. Most of the studies on cultural consumption in the above-mentioned literature focus on analysis of its economic effects, and these findings provide a useful reference for analysis of the theoretical links between expanding cultural consumption and urban air quality. The national cultural consumption pilot policy is an important exploration of expanding cultural consumption in China and has a positive effect on the level of cultural consumption in the pilot cities. In order to obtain the title of “National Cultural Consumption Pilot City”, each city not only needs to meet the basic conditions for application, but also needs to be strictly reviewed by the Ministry of Culture. Because the national cultural consumption pilot has established two types of progressive constraints, “threshold” and “elevated”, the conditions and foundation of cultural consumption in each pilot city are first-class. In addition, the Ministry of Culture has also established a dynamic management mechanismto cancel the pilot status of pilot cities that fail to achieve the expected results. There is no doubt that the national cultural consumption pilot policy provides an opportunity for a quasi-natural experiment to study the impact of expanding cultural consumption on urban air quality. Based on this, this paper uses the national cultural consumption pilot policy based on panel data of 280 Chinese cities from 2006 to 2020,uses the DID model to investigate whether development of cultural consumption with cultural industries at its core can improve urban air quality, and makes an in-depth analysis of its heterogeneity and impact mechanisms.

In this regard, the possible innovations of this paper are: (1) the pollution abatement effect of expanding cultural consumption is empirically assessed by means of the national cultural consumption pilot policy, which not only extends the impact of cultural consumption to the environmental field but also provides some policy incentives for urban environmental protection; (2) this paper examines the heterogeneous impact of expanding cultural consumption on urban air quality from various perspectives, which can help to address environmental pollution challenges in different regions; (3) this paper explores and verifies the moderating effects of government intervention and public participation on emission reduction and pollution reduction effects of expanding cultural consumption, as well as the industrial structure effects and innovation effects of cultural consumption on urban air quality, and analyses the mechanism of expanding cultural consumption on urban air quality from a deeper perspective. 

## 2. Policy Background and Theoretical Assumptions

### 2.1. Policy Background

In 2015, the State Council issued the “Proposal of the Central Committee of the Communist Party of China on Formulating the 13th Five-Year Plan for National Economic and Social Development”, which clearly proposed to “strengthen cultural self-confidence, enhance cultural self-awareness, and accelerate cultural reform and development”.It stressed that promotion of cultural reform and development should put adherence to social benefits in the first place, vigorously develop cultural industries, improve the quality and efficiency of cultural development and make it a pillar industry for national economic development. In 2016, in order to further implement the spirit of expanding and guiding cultural consumption, the Ministry of Culture and the Ministry of Finance decided to launch a nationwide pilot project to guide urban and rural residents in expanding cultural consumption and announced the list of the first batch of 26 national pilot cities for cultural consumption in June of the same year. In 2017, in order to expand the cultural consumption pilot, the two ministries identified a second batch of 19 national pilot cities for cultural consumption. Since the launch of the pilot work, the pilot cities have actively carry out pilot work onexpanding the effective supply of cultural products and services, improving the convenience of cultural consumption, promoting development of cultural tourism, sports and business integration and strengthening promotion of social atmosphere and other aspects, and this work has achieved remarkable results.

### 2.2. Theoretical Hypothesis

(1) The regulatory role of government intervention

The means of intervention by local governments are closely related to the efficiency of resource allocation and the effectiveness of the implementation of national policies. On the one hand, the government’s environmental regulation instruments directly influence the environmental quality of the region and mitigate pollution problems with effective administrative intervention [21]. On the other hand, local governments ensure effective implementation of national policies by stepping up publicity and implementation efforts and formulating targeted policy implementation plans and safeguard mechanisms. If the local government in the pilot cities intervenes less in the policy, it will inevitably weaken the expected benefits of its implementation and fail to effectively promote high-quality development of the cultural industry, leading to low motivation for cultural consumption and weakening the economic and environmental benefits brought about by cultural consumption. Wang and Liao found in their study on the emission reduction effect of carbon markets that government intervention can effectively promote a carbon reduction effect in pilot regions [22]. Shen et al. used a systematic GMM approach to empirically verify the moderating effect of local government intervention on the relationship between monetary policy and liquidity creation in urban commercial banks [23]. Based on this, this paper proposes the following hypothesis.

**Hypothesis** **1 (H1).** *Government intervention positively regulates the role of expanding cultural consumption in improving urban air quality*.

(2) The moderating role of public participation

With the spread of advanced culture and improvement in the public’s level of education, public participation in the process of environmental protection has gradually been recognized and the people have become the main force in construction of ecological civilization. Therefore, the public’s ability to participate in actions to protect the ecological environment is of great significance in solving the problem of environmental pollution. In the process of environmental management, residents can report any damage to the environment through mailboxes, messages on government websites, petitions, etc., and also play a role in monitoring the implementation of environmental policies [24]. In addition, cultural consumption requires participation of all people, and most pilot cities will have initiatives to raise public awareness of spiritual, cultural and environmental consumption responsibilities in their cultural consumption work implementation programs. As the recipients of the policy, active participation of the public can ensure that the pilot policy has the desired effect. Using questionnaires from Jiangsu and Hubei provinces, Chen et al. [25] empirically found that farmers’ participation in the river chief system could significantly improve the management of river ecology. Therefore, the following hypothesis is formulated in this paper.

**Hypothesis** **2 (H2).** *Public participation positively moderates the role of expanding cultural consumption in improving urban air quality*.

(3) Mediation of industrial structure effects and innovation effects

As an important strategic initiative to transform China’s economic development, on the one hand, it promotes growth of the cultural industry and also drives sustainable development of related industries, such as tourism, accommodation, catering, transportation and e-commerce, which is conducive to promoting the cultural industry as a pillar industry of the national economy and improving the advanced level of the industrial structure of the pilot cities. On the other hand, traditional culture, as an important spiritual force in China’s socialist modernization process, contains many innovative ideas for change and can positively contribute to innovation [26]. In addition, industrial structure and innovation are closely linked to air pollution control. Innovation plays an important role in improving environmental quality [5]. Specifically, innovation can reduce the cost of energy use by saving relatively expensive factors of production [27]; i.e., innovation reduces environmental pollution through energy savings [28]; at the same time, technological innovation can improve the environmental performance of firms, thereby enhancing the state of the environment [29]. In studying the relationship between industrial structure and air pollution, most scholars believe that upgrading industrial structure can reduce air pollution levels. Hao et al. found through their study that upgrading the industrial structure is conducive to reducing environmental pollution [30]; Lv and Peng found that development of the tertiary sector plays an important role in reducing environmental pollution using Shanghai as their research target [31]. Based on this, the following hypothesis is formulated in this paper.

**Hypothesis** **3 (H3).** *Expanding cultural consumption can influence air pollution levels in cities through industrial structure effects and innovation effects*.

## 3. Research Design

### 3.1. Baseline Regression Model

In this paper, the cultural consumption pilot is treated as a quasi-natural experiment to study the impact of cultural consumption on urban air quality. Given that the national cultural consumption pilot policy was carried out in batches across the country, the following progressive DID model is constructed to scientifically assess the impact effect of the pilot policy on urban air quality:

(1) $$Y_{\mathrm{it}}=\alpha_{0}+\alpha_{1}{\mathrm{did}}_{\mathrm{it}}+\alpha_{2}X_{\mathrm{it}}+\gamma_{t}+\theta_{i}+{\mathrm{Province}}_{j}\times {\mathrm{Year}}_{t}+\varepsilon_{\mathrm{it}}$$

where the subscripts i, tand j represent city, year and province, respectively; Y is the explained variable in this paper, including industrial SO_2_ emissions per capita and annual average PM2.5 values; did_it_ is a dummy variable for establishment of a national cultural consumption pilot city; X_it_ is a control variable; γ_t_ is a time fixed effect; and θ_i_ is an urban fixed effect; Province_j_ × Year_t_ denotes the interaction effect of province and year, controlling for the effect of factors that vary with province and over time on the estimated results; ε_it_ is a random error term; the estimated coefficient α_1_ measures the average difference in urban air quality before and after the implementation of the pilot policy.

### 3.2. Variable Settings

(1) Explained variables. Urban air quality. The visual manifestation of air quality deterioration is the increase in hazy weather phenomenon, and sulphur dioxide and respirable particulate matter are considered to be the “culprits” of hazy weather. Therefore, this paper uses industrial SO_2_ emissions per capita (*lnpso2*) and average annual PM2.5 (*lnpm*) to measure urban air quality.
(2) Explanatory variables. National cultural consumption pilot policy (*did*). This variable is a dummy variable; i.e., a city takes a value of 1 in the year it becomes a national cultural consumption pilot city and in subsequent years; otherwise, it takes a value of 0.
(3) Control variables. In order to more accurately assess the impact of the national cultural consumption pilot policy on urban air pollution, this paper controls for the following factors that may have an impact on urban air pollution. ① Level of openness to the outside world (*open*). The export trade may be an important cause of China’s current serious air pollution because of the environmental impact caused by packaging and transportation of export products. Therefore, this paper uses the ratio of the total value of imports and exports to the regional GDP to measure the city’s openness to the outside world. ② Level of transport (*road*). Harmful gases from road traffic are the main contributors to hazy weather, so this paper uses the natural logarithm of road area per capita to measure urban traffic levels. ③ Level of financial development (*bank*). Financial development plays an important role in the process of high-quality development of cities; therefore, this paper adopts the proportion of year-end financial institutions’ deposit and loan balances to GDP to measure the level of financial development of cities. ④ Level of internet development (*inter*). The popularity of the Internet has increased the speed of dissemination of factor resources, improved the productivity of enterprises and raised the possibility for reducing pollution; therefore, this paper uses the natural logarithm of the number of international Internet users to measure the level of urban Internet development.
(4) Moderating variables. Government intervention (*gov*): This paper measures government intervention in terms of three instruments of government intervention: science and technology expenditure, fiscal decentralization and environmental regulation. Among them, science and technology expenditure (*tec*) can reflect the strength of government support for scientific research activities, specifically the proportion of government science and technology expenditure to regional GDP. Fiscal decentralization (*fin*) reflects the degree of fiscal autonomy of local governments and is expressed using average municipal fiscal expenditure per capita/(municipal fiscal expenditure per capita + provincial fiscal expenditure per capita + central fiscal expenditure per capita) (fiscal decentralization of municipalities directly under the central government is inscribed using average municipal fiscal expenditure per capita/(municipal fiscal expenditure per capita + central fiscal expenditure per capita)); Environmental regulation (*env*) reflects the degree of importance the government places on the environment and is measured using the share of green space in the municipal area as a percentage of the municipal area. In terms ofpublic participation(*public*), this paper measures public participation in terms of education level, consumer demand and environmental concern. Among them, education level (*edu*) is closely related to the awareness of rights and responsibilities of local residents, which is expressed in this paper as the ratio of the number of university students to the total population. Consumer demand (*con*) can reflect the habits and ability of urban and rural residents to participate in social consumption and is expressed using the natural logarithm of real retail sales of social consumer goods per capita. Environmental concern (*att*) reflects the importance residents attach to the good or bad environment they live in, and drawing on a study by Guo et al. [32], the Baidu Environmental Pollution Search Index was used to portray this.
(5) Intermediate variables. Industrial structure (*ind*), measured using the ratio of tertiary to secondary industries in the gross regional product. Level of innovation (*inn*), expressed using the number of patents granted per 10,000 people.

### 3.3. Data Sources

The PM2.5 data in this paper come from the Socioeconomic Data and Applications Center of Columbia University. Other city-level data involved are mainly from the China City Statistical Yearbook and prefecture-level city statistical yearbooks of previous years, with individual sample gaps filled in by mean interpolation. In order to construct a balanced panel as far as possible, this paper excludes samples with serious missing data (e.g., Lhasa city) and finally conducts an empirical study with 280 prefecture-level cities and 43 national pilot cities for cultural consumption (except county-level cities and autonomous prefectures) from 2006 to 2020. The descriptive statistics of the relevant variables are shown in Table 1.

## 4. Analysis of Study Results

### 4.1. Return to Baseline

Table 2 reports the impact of the national cultural consumption pilot policy on urban air pollution levels, where columns (1) and (3) are regression results controlling for individual, time and province effects without inclusion of control variables. The results show that the effects of the national cultural consumption pilot policy on urban per capita SO_2_ and PM2.5 are both significantly negative at the 1% level. Columns (2) and (4) are the regression results after adding control variables to columns (1) and (3), respectively. The results show that establishment of the policy pilot can still negatively affect urban per capita SO_2_ and PM2.5 levels after controlling for a range of influencing factors, indicating that expanding cultural consumption can effectively reduce air pollutant emissions in local cities. However, we found that the effect of expanding cultural consumption on industrial SO_2_ per capita was greater than that of urban PM2.5, both with and without inclusion of control variables. The reason for this may be that industrial sulphur dioxide is more harmful to the ecological environment than PM2.5, so the government may be more concerned about the impact on SO_2_ in the moderating effect of the pollution-reducing effect of expanding cultural consumption.

### 4.2. Parallel Trends and Dynamic Tests

**Figure 1:**
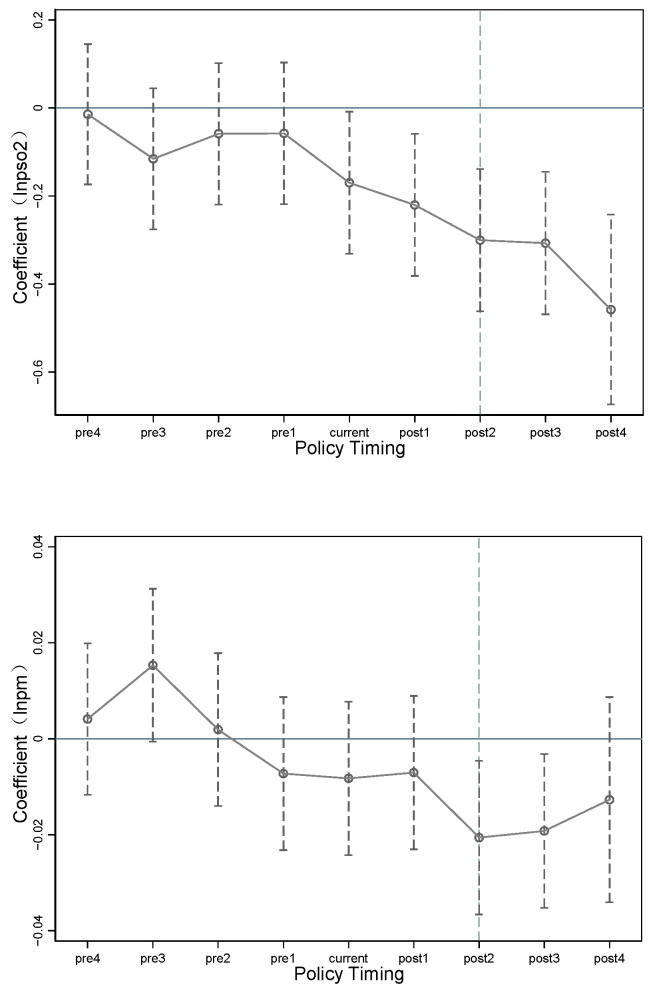
Parallel trend test.

Use of double difference first requires ensuring that the experimental and control groups meet the parallel trend assumption before the policy occurs, i.e., that pilot and non-pilot cities have the same temporal trend prior to implementation of the national cultural consumption pilot policy. As the pilot cities in the experimental group had different years of policy implementation, dummy variables needed to be generated for the current period according to the year in which each pilot city implemented the national cultural consumption pilot policy, as modelled below.

(2) $$Y_{\mathrm{it}}=\alpha_{0}+\overset{4}{\underset{k=-4}{\sum }}\alpha_{k}{\mathrm{did}}_{\mathrm{it}}^{k}+\alpha_{2}X_{\mathrm{it}}+\gamma_{t}+\theta_{i}+{\mathrm{Province}}_{j}\times {\mathrm{Year}}_{t}+\varepsilon_{\mathrm{it}}$$

where i denotes the city and t denotes the year; k is the difference between the year and the year in which the policy was implemented in that pilot city. ${\mathrm{did}}_{\mathrm{it}}^{k}$ is a dummy variable if the year minus the year in which the policy occurred in the city is k, in ${\mathrm{did}}_{\mathrm{it}}^{k}$ = 1; otherwise, it is 0. The control variables are consistent with those in the baseline regression. The regression results are shown in Figure 1. The coefficients on ${\mathrm{did}}_{\mathrm{it}}^{k}$ before the policy were all insignificant and closer to zero, indicating that there was no significant difference in urban air pollution levels between the experimental and control groups before the policy occurred; i.e., the national cultural consumption pilot met the parallel trend hypothesis. In terms of the dynamic effects of expanding cultural consumption, the pilot policy began to have an impact on urban industrial SO_2_ per capita in the year of its establishment, and PM2.5 concentrations began to decrease significantly in the second year after the policy’s implementation, and the impact of the national cultural consumption pilot policy on urban SO_2_ per capita has become increasingly significant over time.

### 4.3. Robustness Tests

#### 4.3.1. PSM-DID

There are two main matching ideas in PSM-DID: one is mixed matching, where panel data are treated as cross-sectional data and matched among experimental and control groups, and the other is period-by-period matching, where individuals in the control group in the same period are selected for matching for the experimental group. Although both methods have certain shortcomings, such as mixed matching where individuals in one period of the experimental group are matched to individuals in other periods of the control group, which would not correctly deal with the time fixed effect, and matching period by period where the control group is unstably matched for each period, both methods are still the better way to conduct research today. Therefore, this paper mitigates the problem of selectivity bias by using mixed matching and period-by-period matching for propensity score matching, respectively, using control variables to represent covariates and using nearest neighbour matching for samples from the experimental and control groups. Table 3 reports the regression results for PSM-DID under both methods. Similar to the baseline regression results, the regression coefficients for the policy dummy variable DID in columns (1) to (4) are significantly negative, indicating that expanding cultural consumption has an abatement effect on urban air pollution, which confirms the robustness of the baseline regression results.

#### 4.3.2. Placebo Test

**Figure 2:**
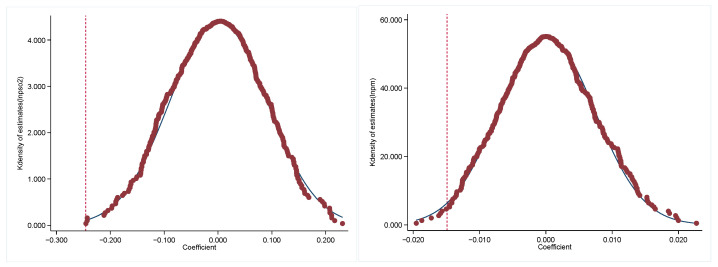
Placebo test.

This paper further uses a placebo test to verify the reliability of the results. This was completed as follows: 43 cities were randomly selected as the “pseudo-experimental group” from the 280 sample cities; i.e., these 43 cities were assumed to be the national pilot cities for cultural consumption while the other cities were used as the control group, and then a year was randomly selected for the “pseudo-experimental group” as the time when the policy took place (pseudo-policy time), and, finally, a “pseudo-policy dummy variable” (interaction term) was generated for regression. This process was repeated 500 times and the regression results up to 500 times were plotted as a distribution of estimated coefficients. The results are shown in Figure 2. The regression coefficient distributions of the models are all close to zero, which shows that the combination of sample times after random sampling has no effect on urban air pollution levels. The sub-vertical dashed lines indicate the estimated coefficients of DID in columns (2) and (4) in Table 2, both of which (−0.2456 and −0.0149) are located in the low tails of the coefficient distributions obtained from random sampling, so the baseline regression results of this paper were further validated.

#### 4.3.3. Removal of Policy Effects

The study period of this paper is 2006–2020, during which other policy events may interfere with the study of this paper, for which this paper excludes the influence of these policy factors in two ways. On the one hand, the empirical results in this paper may be caused by other policy events prior to implementation of the national cultural consumption pilot policy; for this reason, we choose data prior to implementation of the policy (2006–2015) and assume that the pilot policy was implemented uniformly in 2015, which results in a new policy dummy variable, *did2015*, which is substituted into the baseline regression model for regression analysis. The regression results are presented in columns (1) and (2) of Table 4, where we find that the coefficient estimates of *did2015* for both industrial SO_2_ emissions per capita and PM2.5 are not significant, suggesting that the results obtained in this paper are not influenced by other events prior to implementation of the policy. On the other hand, the empirical results of this paper may be related to other events that occurred during the same period or after implementation of the national cultural consumption pilot policy. This paper finds two main policy events that may have an impact on the results of this paper by reading relevant literature and government documents: the ecological restoration city pilot implemented in 2016 and the clean heating pilot policy introduced in 2017. For this reason, we exclude the effects of these two policies separately by excluding the sample. The regression results are presented in columns (3) to (6) of Table 4, and we can find that the results remain robust after excluding the effects of the policies.

#### 4.3.4. Instrumental Variables Approach

Considering that approval of a national cultural consumption pilot is not an entirely exogenous event, selection of a national cultural consumption pilot and urban air quality may have a reciprocal causal relationship and thus endogenous problems. To address this issue, this paper uses an instrumental variables approach to further validate the empirical results of this paper. We choose the number of cultural institutional groups (cinemas and theatres, performing arts groups, public libraries) in each city in 1984 and the city dummy variable of whether or not it was the capital of a historical dynasty (the historical span is from the Qin to Qing dynasties, where there are cases where a temporary capital was established in a particular dynasty, which are not taken into account in this paper) as instrumental variables for the national cultural consumption pilot policy. On the one hand, as mentioned earlier, certain conditions need to be met to be selected as a national cultural consumption pilot, such as the number of cultural consumption sites, and facilities need to rank among the top in the province, so, the more urban cultural institutions and groups, the greater the possibility of being selected as a national cultural consumption pilot. On the other hand, historical dynastic capitals, as the political and cultural centres of their time, represent the advanced culture of a dynasty and have deep cultural heritage and are also more likely to be selected as national pilot cities for cultural consumption. Therefore, these two variables satisfy the condition of relevance as instrumental variables. In terms of exogenous conditions, the city dummy variables for the number of cultural institution groups in each city in 1984 and whether or not they were part of a historical dynastic capital are historical data and do not have an impact on air pollution levels over the sample period. In summary, the urban dummy variables for the number of cultural sites in each city in 1984 and whether they belonged to the capital of an ancient dynasty satisfy the conditions for being instrumental variables.

Table 5 reports the results of the two-stage least squares (2SLS) estimation, with *IV1984WH* and *IVcapital* representing the interaction term between the city dummy variable and the time variable for the number of cultural institution groups at the end of the city year and whether or not they were historical dynastic capitals, respectively. In the first stage regressions, the regression coefficients of the instrumental variables (*IV1984WH*, *Ivcapital*) are all significantly positive and all the results in the table also pass the correlation test for the instrumental variables, indicating that the instrumental variables chosen for this paper are valid. In the second stage regression, the explained variables are industrial sulphur dioxide emissions per capita (*lnpso2*) and PM2.5 (*lnpm*), respectively, and the coefficient of the core explanatory variable (*did*) remains significantly negative, indicating that expanding cultural consumption can significantly curb urban air pollutant emissions, in line with the baseline results and proving the reliability of the previous study’s findings.

#### 4.3.5. Breakpoint Inspection

**Figure 3:**
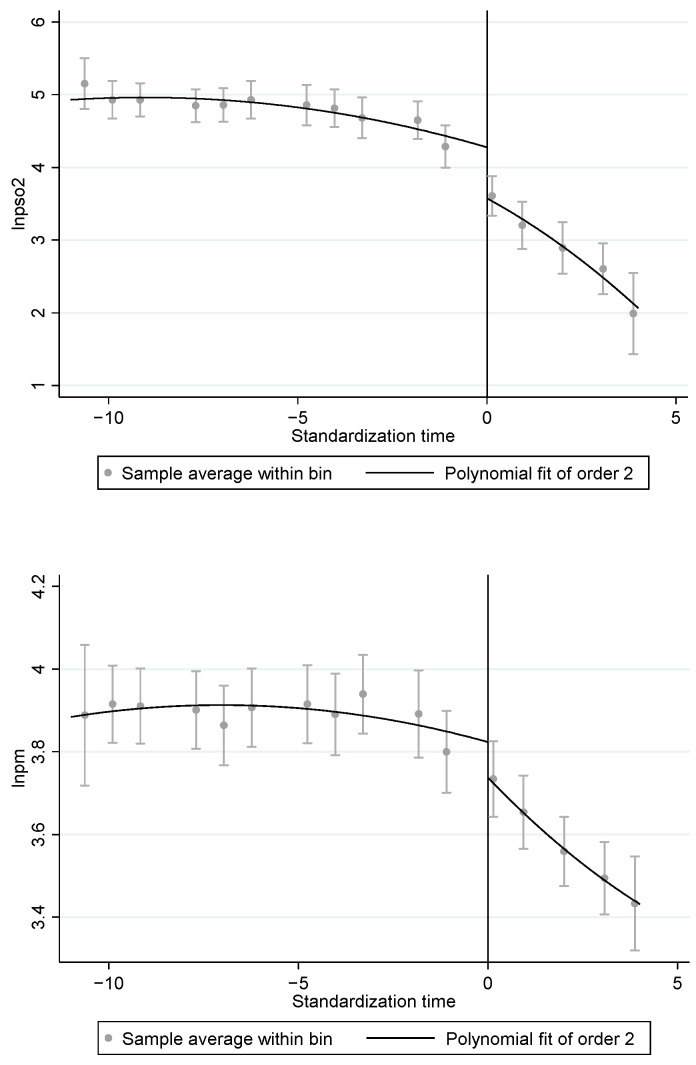
Breakpoint diagram.

We drew on the research idea of the RD method by subtracting the year in which the pilot city was located from the year in which it was identified as a national cultural consumption pilot to examine whether there was a discontinuous change in air quality at the breakpoint when the policy occurred, and the results are shown in Figure 3 (The optimal order of the execution variable is selected as second order according to the AIC and BIC judging criteria). PM2.5 emission concentrations and industrial SO_2_ emissions per capita show a downward jump at the time of the policy occurrence at the policy, and the overall emission trend after the policy occurrence is significantly lower than before the policy occurrence, indicating that the policy effects identified based on the DID model are real.

#### 4.3.6. Other Robustness Tests

(1) Substitution of explained variables. The Air Quality Index provides a comprehensive picture of the level of air pollution in an area. For this reason, the explained variable is replaced with the air quality index (*lnaqi*) in this paper. The data are obtained from the China Air Quality Online Inspection and Analysis Platform, which has been publishing monthly air quality data for each city since December 2013. Given that this paper studies an annual indicator, we take the monthly data for each city as the annual average value and use it as the annual air quality index for each city. The regression results are presented in column (1) of Table 6, and the results show that the regression coefficient of DID is negative at the 1% significance level, which is consistent with the findings obtained from the baseline regression and proves the robustness of the empirical results.
(2) Time trend term controlling the administrative level of the city. We added a dummy variable for the administrative level of the city (municipality directly under the central government, provincial capital city) to the baseline model with an interaction term for the time trend term to exclude the effect of fixed characteristics associated with the administrative level of the city on urban air pollution levels. The regression results are presented in columns (2) and (3) of Table 6, which show that the regression coefficient of DID remains significantly negative after controlling for the administrative level of the city, demonstrating the robustness of the empirical results.
(3) With neighbouring cities in the province as the control group. In this paper, cities that are geographically bordering the provinces where the national cultural consumption pilot cities are located are selected as the control group and then re-tested to ensure that the sample characteristics of the experimental and control groups are as similar as possible. The regression results are presented in columns (4) and (5) of Table 6 and show that the regression coefficient for DID remains significantly negative after re-screening the control group, demonstrating the robustness of the empirical results.
(4) Select issue to handle. Identification of a national cultural consumption pilot may be related to the air quality of the city before the policy took place; to rule out this possibility, we used data from before the pilot policy took place (2015) as a sample, with industrial sulphur dioxide emissions per capita (*lnpso2*) and PM2.5 (*lnpm*) as explanatory variables and whether or not it was a national cultural consumption pilot (*treat*) as the explained variable, respectively, and conducted regression analysis. The regression results in columns (6) and (7) of Table 6 show that the cities that later became national cultural consumption pilots were not related to their air quality levels prior to the pilots.

**Table 6:** Other robustness tests.

Variables	*Lnaqi*	*Lnpso2*	*Lnpm*	*Lnpso2*	*Lnpm*	*Treat*	*Treat*
	(1)	(2)	(3)	(4)	(5)	(6)	(7)
*did*	−0.0205 ***	−0.1134 **	−0.0168 ***	−0.2730 ***	−0.0120 ***		
	(0.008)	(0.050)	(0.005)	(0.045)	(0.004)		
*lnpso2*						0.0133	
						(0.028)	
*lnpm*							−0.0123
							(0.111)
*_cons*	4.2628 ***	5.8299 ***	3.8400 ***	5.8841 ***	3.9930 ***	−1.4997 ***	−1.3648 **
	(0.106)	(0.650)	(0.065)	(0.633)	(0.059)	(0.444)	(0.546)
*Control*	Yes	Yes	Yes	Yes	Yes	Yes	Yes
*Year*	Yes	Yes	Yes	Yes	Yes	No	No
*City*	Yes	Yes	Yes	Yes	Yes	No	No
*Province*	Yes	Yes	Yes	Yes	Yes	Yes	Yes
*N*	1960	4200	4200	2205	2205	280	280

Note: standard errors are in parentheses in the table; *, ** and *** indicate significant at the 10%, 5% and 1% levels, respectively.

### 4.4. Heterogeneity Analysis

#### 4.4.1. Urban Location Heterogeneity Test

The Qinling–Huaihe line is the geographical boundary between the north and the south of China, and there are certain differences in climatic conditions, living customs and geographical features between the northern and southern sides of this line. Compared to southern cities, northern cities adopt central heating in winter, which undoubtedly increases the level of air pollution in northern cities. Based on this, this paper empirically tests whether the effect of expanding cultural consumption on air pollution intensity differs along the Qinling–Huaihe line. The regression results in Table 7 show that establishment of pilot cities north of the Qinling–Huaihe line has a stronger pollution abatement effect compared to cities south of the Qinling–Huaihe line. The main reason may be that the air quality in the cities north of the Qinling–Huaihe line is relatively poor. When the pilot policy is implemented, the government will put more effort into managing the urban environment and develop a more reasonable implementation plan to ensure the smooth implementation of the policy.

#### 4.4.2. Heterogeneity Analysis of Urban Resource Endowments

Areas with resource endowments tend to rely more on their own resources to drive economic and social development, and their industrial structure and economic development approach will be different compared to other cities. To examine whether the emission-reducing and pollution-reducing effects of cultural consumption show variability according to the resource endowment of cities, this paper divides the sample into resource-based and non-resource-based cities. The specific regression results are shown in Table 7, which shows that establishment of national pilot cities for cultural consumption can effectively reduce air pollution levels in non-resource-based cities, but the pollution reduction effect on resource-based cities is not significant. The main reason may be that the development achieved by resource-based cities over a long period of time is mainly dependent on resource-based industries, and the industrial structure is characterised by a prominent reliance on energy, under which conditions path dependence and lock-in effects tend to form, which is not conducive to the effect of expanding cultural consumption pollution reduction.

## 5. Mechanism Testing

### 5.1. Testing the Moderating Effect of Government Intervention and Public Participation

Successful implementation of the policy cannot be achieved without strict control of the local government; thus, government intervention may have a moderating effect on the pollution reduction effect of cultural consumption in the pilot areas. In order to test the moderating effect of government intervention on the pollution reduction and emission reduction effect of expanding cultural consumption, the following model is set up in this paper:

(3) $$Y_{\mathrm{it}}=\alpha_{0}+\alpha_{1}{\mathrm{did}}_{\mathrm{it}}+{\mu \mathrm{did}}_{\mathrm{it}}\times {\mathrm{gov}}_{\mathrm{it}}+\beta_{g}{\mathrm{gov}}_{\mathrm{it}}+\alpha_{2}X_{\mathrm{it}}+\gamma_{t}+\theta_{i}+{\mathrm{Province}}_{j}\times {\mathrm{Year}}_{t}+\varepsilon_{\mathrm{it}}$$

where gov_it_ is the relevant indicator measuring government intervention, specifically science and technology expenditure (*tec*), fiscal decentralization (*fin*) and environmental regulation (*env*), and the coefficient μ is the key variable for determining the existence of the moderating effect of government intervention, with the other signs having the same significance as in the baseline regression model.

Table 8 shows the relevant regression results. It can be seen that government intervention has a positive moderating effect on the sulphur reduction effect of cultural consumption regardless of which means of government administrative intervention, from science and technology expenditure, fiscal decentralization or environmental regulation, and, therefore, hypothesis H1 is tested. When administrative intervention is measured in terms of technology expenditure, the coefficient μ is negative but not significant for PM2.5, indicating that the government cannot influence the haze reduction effect of expanding cultural consumption by means of increased technology expenditure. When administrative intervention is measured in terms of fiscal decentralization and environmental regulation, the coefficient μ remains insignificant for PM2.5, which implies that government intervention has not yet played a role in the haze reduction effect of the pilot policy, which explains why the sulphur reduction effect of the pilot policy is greater than its haze reduction effect in the previous section. In summary, the pollution-reducing effect of cultural consumption is influenced by government intervention; i.e., the stronger the administrative intervention in the implementation of the policy, the greater the effect of cultural consumption on urban air pollution. In addition, government intervention has a limited impact on the pollution reduction effect of cultural consumption as government intervention has failed to effectively regulate the haze reduction effect of cultural consumption and other ways exist to significantly contribute to the pollution reduction and emission reduction effect of cultural consumption.

Given that there are other ways to influence the pollution-reducing effects of expanding cultural consumption, is this way related to public participation? To test this conjecture, the following model was set up:

(4) $$Y_{\mathrm{it}}=\alpha_{0}+\alpha_{1}{\mathrm{did}}_{\mathrm{it}}+{\mu \mathrm{did}}_{\mathrm{it}}\times {\mathrm{public}}_{\mathrm{it}}+\beta_{g}{\mathrm{public}}_{\mathrm{it}}+\alpha_{2}X_{\mathrm{it}}+\gamma_{t}+\theta_{i}+{\mathrm{Province}}_{j}\times {\mathrm{Year}}_{t}+\varepsilon_{\mathrm{it}}$$

where public_it_ is the relevant indicator measuring public participation, specifically education level (*edu*), consumption level (*con*) and environmental concern (*att*), and the coefficient μ is the key variable to determine the existence of the moderating effect of public participation, with the other signs having the same meaning as in the baseline regression model.

Table 9 presents the results of the regressions. The interaction term between the policy dummy and education level is significantly negative for both industrial SO_2_ per capita and PM2.5, so, the more educated the public is, the more significant the pollution reduction effect is in the pilot cities, and hypothesis H2 is tested. When measuring public participation in terms of consumption levels, the regression coefficient of its interaction term remains significantly negative and the findings of this paper remain unchanged. When the moderating effect of public participation from the perspective of environmental concern is analysed, the regression coefficient of the interaction term is still significantly negative at the 10% level and the findings remain unchanged.

In summary, the pilot cultural consumption policy is effective in improving air quality levels in cities.In addition, government intervention and public participation can effectively enhance the pollution-reducing effects of the pilot cultural consumption policy.

### 5.2. Intermediary Effects Test

Based on the analysis of the previous research hypothesis, this paper argues that cultural consumption can improve the air quality level in cities through two channels: industrial structure and innovation. Therefore, inorderto further test research hypothesis H4, this paper draws on the research of Baron and Kenny [33] to empirically test the above two channels of action by means of a mediating effects test. The model is set up as follows:

(5) $$M_{\mathrm{it}}=\alpha_{0}+\alpha_{1}{\mathrm{did}}_{\mathrm{it}}+\alpha_{2}X_{\mathrm{it}}+\gamma_{t}+\theta_{i}+{\mathrm{Province}}_{j}\times {\mathrm{Year}}_{t}+\varepsilon_{\mathrm{it}}$$

(6) $$Y_{\mathrm{it}}=\alpha_{0}+\alpha_{1}{\mathrm{did}}_{\mathrm{it}}+{\eta M}_{\mathrm{it}}+\alpha_{2}X_{\mathrm{it}}+\gamma_{t}+\theta_{i}+{\mathrm{Province}}_{j}\times {\mathrm{Year}}_{t}+\varepsilon_{\mathrm{it}}$$

where M_it_ is the relevant mediating variable, specifically industry structure (*ind*), innovation level (*inn*), coefficient $\alpha_{1}$ and coefficient η are the key variables to determine the existence of the mediating effect, and the other symbols have the same meaning as in the baseline regression model.

The results of the mediating effect of the industrial structure are shown in columns (1) to (3) of Table 10. It can be seen that the estimated coefficient of the impact of the national cultural consumption pilot policy on the industrial structure is positive and significant at the 1% level, indicating that implementation of the national cultural consumption pilot policy can adjust the local industrial structure and promote the advanced development of industrial structure, and the regression coefficients of the core explanatory variables (*did*) and industrial structure (*ind*) on urban per capita of industrial SO_2_ and PM2.5 are both negative and significant at the 1% level, indicating the existence of the mediating effect of industrial structure. The results of the mediating effect of innovation are presented in columns (4) to (6) of Table 10, which show that the national pilot policy on cultural consumption can significantly promote the level of innovation in cities and that the regression coefficients of the core explanatory variables (*did*) and the level of innovation (*inn*) on urban industrial SO_2_ per capita and PM2.5 are both negative, indicating the existence of the mediating effect of innovation. In summary, expanding cultural consumption can reduce urban air pollution levels by both restructuring local industries and increasing the level of urban innovation, and hypothesis H3 is tested.

## 6. Conclusions and Discussion

### 6.1. Conclusions

Based on panel data of 280 cities in China from 2006 to 2020, this paper considers the national cultural consumption pilot policy implemented in batches starting from 2016 as a quasi-natural experiment and constructs a progressive DID model to empirically test the inhibitory effect of cultural consumption on urban air pollution, as well as the heterogeneity analysis of this effect and the principle of action. The findings show that: (1) the effect of the cultural consumption pilot policy on air quality is real and its sulphur reduction effect is stronger than its effect on PM2.5, and this conclusion still holds after a series of robustness tests. (2) Heterogeneity analysis shows that the pollution-reducing effect of expanding cultural consumption is stronger in cities north of the Qinling–Huaihe line; in terms of resource endowment, non-resource cities can reduce air pollution through expanding cultural consumption, while the pollution-reducing effect of expanding cultural consumption in resource-based cities is not significant. (3) The mechanism of action test shows that government intervention and public participation can positively regulate the impact of expanding cultural consumption on urban air pollution and that expanding cultural consumption can reduce urban air pollution mainly through two ways: adjusting the structure of local industries and improving the level of urban innovation.

Based on the above findings, this paper makes the following policy recommendations. (1) Establishment of a sound government guidance mechanism. Local governments should strengthen planning and policy guidance for cultural consumption and make comprehensive use of financial power to guide synergistic development of cultural industries and environmental protection, broaden the financing channels for the cultural industry, continuously increase capital investment for development of the cultural industry and guide sustainable development of the cultural industry. (2) Consolidate the mass foundation for construction of ecological civilization and maximize the synergistic contribution of public participation. The spiritual attributes of cultural products are actively used to transform environmental protection into social consensus, enrich people’s spiritual and civilized lives and stimulate construction of ecological civilization. In practice, this can be accomplished by building eco-cultural museums, showing public service announcements, films and documentaries on protecting the environment and hosting various eco-themed exhibitions to spread environmental knowledge and actively advocate ecological values and consumption so that people can participate in construction of ecological civilization, practice a low-carbon lifestyle and establish the concept of ecological civilization. (3) Improve innovation and create cultural enterprises or industries with local brands. Local governments should improve the innovation system and mechanism, pay attention to protection of intellectual property rights, follow the trends of the times, adapt to the modern society’s pursuit of new, beautiful and environmentally friendly consumption characteristics, integrate innovation into all aspects of cultural consumption and accelerate cultivation of new markets that combine novel culture and environmental protection. (4) Adhere to the principle of adapting to local conditions. Development of cultural consumption has different degrees of pollution reduction and emission reduction effects in cities with different geographical locations and resource endowments. Therefore, the government should formulate targeted policies and plans to promote cultural consumption according to the city’s own characteristics and avoid blindly copying the experiences of other regions.

### 6.2. Discussion

It is noteworthy that not many studies have been conducted on the environmental impact of cultural consumption. Vicente-Molina et al. argue that cultural engagement increases the probability of emergence of pro-environmental behaviours in people [34]. Agovino et al. [35] have found that cultural consumption can enhance people’s motivation for pro-environmental behaviour and concluded that cultural policies can play a relevant role in addressing environmental issues. It is easy to see that the above studies mainly consider that cultural consumption improves the environment by influencing people’s behaviour and do not examine the impact of cultural consumption from a socio-economic perspective. It is important to emphasize that this paper explores the role of expanding cultural consumption in enhancing urban air quality from a macroeconomic perspective. Further, this paper also finds a significant role of public participation and government intervention in terms of the impact of expanding cultural consumption on air quality.

The significance of this paper is both explanatory and illuminating: on the one hand, while the logic of the topic of expanding cultural consumption to improve air quality is not difficult, the difficulty lies in the empirical evidence because it is not easy to perfectly portray cultural consumption and address endogeneity as much as possible. This paper uses the national cultural consumption pilot policy to conduct a study that largely increases the explanatory power of the topic that expanding cultural consumption can improve air quality. On the other hand, this paper emphasizes the importance of government intervention, which has not yet fully played its positive regulatory role and enlightens policymakers to strengthen the institutional design and arrangement in the pilot policy.

## Figures and Tables

**Table 1:** Descriptive statistics of variables.

Variables	Obs.	Mean	Std. Dev.	Min	Max
*lnpso2*	4200	4.2508	1.3110	−5.0345	7.8382
*lnpm*	4200	3.7317	0.3476	2.4522	4.6870
*open*	4200	0.1924	0.3349	0.0000	3.4988
*road*	4200	1.1009	0.8289	−5.5662	3.9870
*bank*	4200	2.2799	1.1618	0.5600	21.3015
*inter*	4200	6.0696	1.1446	−1.4271	9.5639

**Table 2:** Baseline regression results.

Variables	*Lnpso2*		*Lnpm*	
	(1)	(2)	(3)	(4)
*did*	−0.3152 ***	−0.2456 ***	−0.0157 ***	−0.0149 ***
	(0.045)	(0.045)	(0.004)	(0.004)
*open*		0.4888 ***		0.0196 ***
		(0.069)		(0.007)
*road*		0.1612 ***		0.0004
		(0.029)		(0.003)
*bank*		−0.0324 *		−0.0022
		(0.018)		(0.002)
*inter*		0.0521 *		−0.0032
		(0.029)		(0.003)
*_cons*	6.9348 ***	6.0100 ***	3.8349 ***	3.8374 ***
	(0.637)	(0.653)	(0.062)	(0.065)
*Year*	Yes	Yes	Yes	Yes
*City*	Yes	Yes	Yes	Yes
*Province*	Yes	Yes	Yes	Yes
*N*	4200	4200	4200	4200

Note: standard errors are in parentheses in the table; *, ** and *** indicate significant at the 10%, 5% and 1% levels, respectively.

**Table 3:** PSM-DID regression results.

Variables	Mixed Matching		Period-by-Period Matching	
	*Lnpso2*	*Lnpm*	*Lnpso2*	*Lnpm*
*did*	−0.2424 ***	−0.0145 ***	−0.1459 **	−0.0110 *
	(0.045)	(0.004)	(0.060)	(0.006)
*_cons*	6.0260 ***	3.8364 ***	3.2762 ***	3.4555 ***
	(0.659)	(0.066)	(0.517)	(0.053)
*Control*	Yes	Yes	Yes	Yes
*Year*	Yes	Yes	Yes	Yes
*City*	Yes	Yes	Yes	Yes
*Province*	Yes	Yes	Yes	Yes
*N*	4114	4114	1490	1490

Note: standard errors are in parentheses in the table; *, ** and *** indicate significant at the 10%, 5% and 1% levels, respectively.

**Table 4:** Regression results after excluding other policy factors.

Variables	*Lnpso2*	*Lnpm*	*Lnpso2*	*Lnpm*	*Lnpso2*	*Lnpm*
	(1)	(2)	(3)	(4)	(5)	(6)
*did2015*	−0.1052	−0.0119				
	(0.071)	(0.008)				
*did*			−0.2807 ***	−0.0091 *	−0.2302 ***	−0.0137 ***
			(0.053)	(0.005)	(0.049)	(0.005)
*_cons*	5.6096 ***	3.6766 ***	6.0845 ***	3.8576 ***	6.0297 ***	3.7748 ***
	(0.569)	(0.063)	(0.633)	(0.063)	(0.654)	(0.066)
*Control*	Yes	Yes	Yes	Yes	Yes	Yes
*Year*	Yes	Yes	Yes	Yes	Yes	Yes
*City*	Yes	Yes	Yes	Yes	Yes	Yes
*Province*	Yes	Yes	Yes	Yes	Yes	Yes
*N*	2800	2800	3435	3435	3630	3630

Note: standard errors are in parentheses in the table; *, ** and *** indicate significant at the 10%, 5% and 1% levels, respectively.

**Table 5:** Results of the test based on two-stage least squares.

Variables	First Stage Regressions	Second Stage Regressions	
	*Did*	*Lnpso2*	*Lnpm*
*IV1984WH*	0.0108 ***		
	(0.001)		
*IVcapital*	0.0233 ***		
	(0.004)		
*did*		−1.6776 ***	−0.0666 ***
		(0.184)	(0.025)
*Control*	Yes	Yes	Yes
*Year*	Yes	Yes	Yes
*City*	Yes	Yes	Yes
*Kleibergen–Paap LM*	173.866 ***		
*Kleibergen–Paap Wald F*	127.006 [19.93]		
*Hansen J Test*		0.4602	0.1087
*N*	3300	3300	3300

Note: standard errors are in parentheses in the table; *, ** and *** indicate significant at the 10%, 5% and 1% levels, respectively.

**Table 7:** Analysis of heterogeneity.

Variables	Northern Cities		Southern Cities		Non-Resource-Based Cities		Resource-Based Cities	
	*Lnpso2*	*Lnpm*	*Lnpso2*	*Lnpm*	*Lnpso2*	*Lnpm*	*Lnpso2*	*Lnpm*
*did*	−0.3226 ***	−0.0157 **	−0.1525 **	−0.0125 **	−0.3083 ***	−0.0184 ***	−0.0015	−0.0022
	(0.062)	(0.007)	(0.064)	(0.006)	(0.057)	(0.005)	(0.085)	(0.009)
*_cons*	4.3636 ***	3.9291 ***	5.7309 ***	3.7361 ***	5.3882 ***	3.8453 ***	4.3356 ***	3.6647 ***
	(0.317)	(0.035)	(0.685)	(0.059)	(0.685)	(0.061)	(0.365)	(0.040)
*Control*	Yes	Yes	Yes	Yes	Yes	Yes	Yes	Yes
*Year*	Yes	Yes	Yes	Yes	Yes	Yes	Yes	Yes
*City*	Yes	Yes	Yes	Yes	Yes	Yes	Yes	Yes
*Province*	Yes	Yes	Yes	Yes	Yes	Yes	Yes	Yes
*N*	1905	1905	2295	2295	2505	2505	1695	1695

Note: standard errors are in parentheses in the table; *, ** and *** indicate significant at the 10%, 5% and 1% levels, respectively.

**Table 8:** Tests of moderating effects of government intervention.

Variables	*Lnpso2*			*Lnpm*		
	(1)	(2)	(3)	(4)	(5)	(6)
*did*	−0.1518 **	0.5744 **	−0.1042 *	−0.0116 *	−0.0061	−0.0117 **
	(0.067)	(0.234)	(0.057)	(0.007)	(0.023)	(0.006)
*did × tec*	−17.2753 *			−0.4594		
	(10.383)			(1.030)		
*did × fin*		−2.3887 ***			−0.0257	
		(0. 680)			(0.068)	
*did × env*			−1.9518 ***			−0.0453
			(0.493)			(0.049)
*_cons*	5.9210 ***	4.7252 ***	5.7150 ***	3.8334***	3.8240 ***	3.8283 ***
	(0.654)	(0.692)	(0.658)	(0.065)	(0.069)	(0.065)
*Control*	Yes	Yes	Yes	Yes	Yes	Yes
*Year*	Yes	Yes	Yes	Yes	Yes	Yes
*City*	Yes	Yes	Yes	Yes	Yes	Yes
*Province*	Yes	Yes	Yes	Yes	Yes	Yes
*N*	4200	4200	4200	4200	4200	4200

Note: standard errors are in parentheses in the table; *, ** and *** indicate significant at the 10%, 5% and 1% levels, respectively.

**Table 9:** Tests of the moderating effect of public participation.

Variables	*Lnpso2*			*Lnpm*		
	(1)	(2)	(3)	(4)	(5)	(6)
*did*	0.0206	3.8879 ***	−0.0467	−0.0037	0.2098 **	−0.0052
	(0.075)	(0.955)	(0.082)	(0.008)	(0.095)	(0.009)
*did* × *edu*	−0.0593 ***			−0.0024 *		
	(0.013)			(0.001)		
*did × con*		−0.4081 ***			−0.0222 **	
		(0.094)			(0.009)	
*did × att*			−0.0022 *			−0.0002 *
			(0.001)			(0.000)
*_cons*	6.0835 ***	5.0296 ***	3.3977 ***	3.8495 ***	3.7909 ***	3.7492 ***
	(0.655)	(0.721)	(0.378)	(0.065)	(0.072)	(0.042)
*Control*	Yes	Yes	Yes	Yes	Yes	Yes
*Year*	Yes	Yes	Yes	Yes	Yes	Yes
*City*	Yes	Yes	Yes	Yes	Yes	Yes
*Province*	Yes	Yes	Yes	Yes	Yes	Yes
*N*	4200	4200	2760	4200	4200	2760

Note: standard errors are in parentheses in the table; *, ** and *** indicate significant at the 10%, 5% and 1% levels, respectively.

**Table 10:** Intermediary effects test.

Variables	*Ind*	*Lnpso2*	*Lnpm*	*Inn*	*Lnpso2*	*Lnpm*
	(1)	(2)	(3)	(4)	(5)	(6)
*did*	0.0450 ***	−0.2322 ***	−0.0143 ***	5.9811***	−0.1577 ***	−0.0113 **
	(0.017)	(0.044)	(0.004)	(0.421)	(0.046)	(0.005)
*ind*		−0.2985 ***	−0.0121 ***			
		(0.044)	(0.004)			
*inn*					−0.0147 ***	−0.0006 ***
					(0.002)	(0.000)
*_cons*	−0.3245	5.9131 ***	3.8335 ***	0.0009	6.1475 ***	3.8429 ***
	(0.251)	(0.649)	(0.065)	(0.001)	(0.647)	(0.065)
*Control*	Yes	Yes	Yes	Yes	Yes	Yes
*Year*	Yes	Yes	Yes	Yes	Yes	Yes
*City*	Yes	Yes	Yes	Yes	Yes	Yes
*Province*	Yes	Yes	Yes	Yes	Yes	Yes
*N*	4200	4200	4200	4200	4200	4200

Note: standard errors are in parentheses in the table; *, ** and *** indicate significant at the 10%, 5% and 1% levels, respectively.

## Data Availability

The data in this study can be found in publicly available databases including China City Statistical Yearbook and China Statistical Yearbook (https://data.cnki.net/yearBook?type=type&code=A), as well as Chinese Research Data Services Platform (www.cnrds.com), Socioeconomic Data and Applications Center of Columbia University (https://beta.sedac.ciesin.columbia.edu/), and Baidu Index (https://index.baidu.com/v2/index.html#/).
